# Efficacy of Bacteriophages Against *Staphylococcus aureus* Isolates from Bovine Mastitis

**DOI:** 10.3390/ph13030035

**Published:** 2020-02-26

**Authors:** Isabel Titze, Tatiana Lehnherr, Hansjörg Lehnherr, Volker Krömker

**Affiliations:** 1 Department of Bioprocess Engineering and Microbiology, University of Applied Sciences and Arts Hannover, D-30453 Hannover, Germany; isabel.titze@tiho-hannover.de; 2 Phage Technology Center GmbH, D-59199 Bönen, Germany; t.lehnherr@ptc-phage.com (T.L.); h.lehnherr@ptc-phage.com (H.L.); 3 Section for Production, Nutrition and Health, Department of Veterinary and Animal Sciences, Faculty of Health and Medical Sciences, University of Copenhagen, DK-1870 Frederiksberg C, Denmark

**Keywords:** bacteriophage mixture, phage therapy, lytic phage, dairy, bovine mastitis

## Abstract

The lytic efficacy of bacteriophages against *Staphylococcus aureus* isolates from bovine milk was investigated in vitro, regarding possible applications in the therapy of udder inflammation caused by bacterial infections (mastitis). The host range of sequenced, lytic bacteriophages was determined against a collection of 92 *Staphylococcus* (*S.*) *aureus* isolates. The isolates originated from quarter foremilk samples of clinical and subclinical mastitis cases. A spot test and a subsequent plaque assay were used to determine the phage host range. According to their host range, propagation and storage properties, three phages, STA1.ST29, EB1.ST11, and EB1.ST27, were selected for preparing a bacteriophage mixture (1:1:1), which was examined for its lytic activity against *S. aureus* in pasteurized and raw milk. It was found that almost two thirds of the isolates could be lysed by at least one of the tested phages. The bacteriophage mixture was able to reduce the *S. aureus* germ density in pasteurized milk and its reduction ability was maintained in raw milk, with only a moderate decrease compared to the results in pasteurized milk. The significant reduction ability of the phage mixture in raw milk promotes further in vivo investigation.

## 1. Introduction

Dealing with udder health disorders, including clinical and subclinical inflammation of the bovine mammary gland (mastitis), is one of the most important issues in the dairy industry. Mastitis leads to economic losses due to discarded milk, reduced milk production and a decrease in milk quality. Furthermore, it is a reason for culling chronically infected dairy cows, increasing costs due to additional labor, medication, and veterinary services and it impairs animal welfare [1,2,3].

*Staphylococcus* (*S*.) *aureus* is the most frequently isolated member of the contagious mastitis pathogens (major pathogen) [4,5,6]. There is a high risk of transmission of *S. aureus* between animals, especially during the milking process, and the pathogen has the ability to persist as a subclinical infection of the bovine udder. Important virulence factors of *S. aureus*-isolates enable their adhesion to epithelial cells, encapsulation, the formation of micro abscesses and the formation of biofilms, impeding treatment by antimicrobial agents and promoting a chronic progression [7,8]. Thus, an adequate treatment of *S. aureus* mastitis requires hygiene and special management routines, with three measures being identified for *S. aureus* intramammary infections (IMIs): a) separating the positively tested animals, b) culling the chronically infected animals and c) undertaking adequate hygiene and treatment measures [5,9]. Conventional treatment of *S. aureus* IMIs in the lactation and in the dry period requires the use of antimicrobial agents [10]. While dry cow therapy leads to cure rates of up to 78% for *S. aureus* mastitis [11,12], the bacteriological cure (BC) rates for treatment during lactation are considerably lower for *S. aureus* compared to other pathogens [4,13,14]. The reported cure rates for a lactation treatment of *S. aureus* mastitis vary between studies and depend on animal, pathogen and treatment factors as well as study design [5]. However, the cure rates for an antimicrobial treatment of *S. aureus* mastitis during lactation are generally considered unsatisfactory [13,14,15]. They decrease with high somatic cell counts (SCC), higher parity of cows, ß-lactamase-positive *S. aureus* infections and require an extended antimicrobial therapy to improve BC [4]. Linder et al. [14] investigated the BC rate of chronic subclinical *S. aureus* infections under treatment with marbofloxacin and cephalexin, and reported a remarkably low BC rate of 21.9% for treatment during lactation. This underlines a low accessibility for an antimicrobial treatment of *S. aureus* IMIs in the milking period and directs the attention to the development of resistance against the antimicrobial agents.

Although the resistance situation of antimicrobial agents for mastitis therapy in Germany is still moderate, there are studies describing the occurrence of resistant *S. aureus* isolates in dairy herds in addition to frequently occurring resistance to ß-lactam antibiotics [10,16,17]. The examination of Kreausukon et al. [17] reports the isolation of “Livestock-associated Methicillin resistant *S. aureus”* (LA-MRSA) isolates from bulk tank milk of German dairy herds with resistance especially against tetracycline, clindamycin and erythromycin. Spohr et al. [16] found a remarkable spread of MRSA of the *spa*-type t011 in three dairy herds in Southwest Germany. The fact that each application of antimicrobial agents promotes the emergence of resistance in bacterial isolates and an increasing awareness of resistance development, requires an overall reduction in the use of antimicrobial agents in human and veterinary medicine [2,18,19]. This emphasizes the need for alternative solutions for the treatment of bacterial infections, including conventional mastitis treatment. At this point, the therapeutic use of bacteriophages could be such an alternative for treating bacterial infections in animals as already successfully demonstrated in several animal models [20,21,22].

Bacteriophages (phages) are viruses that solely use prokaryotic cells for propagation. These highly host-specific agents occur ubiquitously in nature and can be isolated from the environment with comparatively little time and effort. After binding to specific structures on the host cell surface, the phage-genome is injected into the bacterial cell. Following replication of phage DNA or RNA and assembly of the viruses, phage-encoded enzymes lead to the lysis of the bacterial cell (lytically infecting phages) [23]. This results in the release of next generation phages and to an exponential growth of the phage population [24]. Thereby, the proliferation of phages occurs only as long as an adequately high bacterial concentration is present at the target site. This characteristic offers a possible advantage of so-called “self-limitation” for a therapeutic approach [22]. 

Bacteriophages can be grouped into four categories according to their infection strategies, based on the following criteria: a) does virion release occur (e.g., productive infection versus lysogeny of the cell when the phage genome is incorporated into the host cell genome (so-called prophage)), b) how does phage release occur (lytic or chronic), c) do the phage exhibit a certain degree of lysogenic cycles due to their genetics (distinction between temperate and non-temperate phage). For a detailed summary of these terms, see the 2016 article by Hobbs and Abedon [23]. A classification of phages according to their infection strategies, as advised by the authors, indicates I) lytic and non-temperate phages, II) chronic and non-temperate phages, III) lytic and temperate phages, and IV) chronic and temperate phages [23]. For therapeutic purposes, “professionally lytic” phages (category I), i.e., those that are obligately lytic, do not belong to the temperate phages and are not recent lytic mutants of the temperate phages, should be used [23,25].

Genes that code for toxins are important regarding the use of phages in therapy. These are potentially transferred by temperate bacteriophages (lysogenic conversion) [26]. The screening of phage genomes for the absence of lysogenic potential and toxin-coding genes are further steps that can be carried out before phages are used as therapeutic agents [25,27].

A number of studies already investigated the bactericidal activity of bacteriophages against *S. aureus* in milk, some of them with regard to food safety and the use of phages as biocontrol agents and others with regard to the use of phages as an alternative therapeutic agent to combat bacterial infections of the bovine udder. Milk represents a complex medium composed of different components like lipids, milk proteins (caseins, whey proteins) and an own bacterial flora [28]. Concerning this, the investigation of phages as an alternative treatment method for *S. aureus* mastitis in cattle requires an investigation of the bactericidal activity of phages in bovine milk. It was shown in former studies that a previous heat treatment (UHT > pasteurized) and the skimming of milk have a positive effect on the bactericidal activity of the phages. The authors assumed an inhibition of the ability of the phages to bind to their host cells in raw milk and mainly suggested lipids and whey proteins to be responsible for this [29,30]. The previous studies showed a decrease in phage titer or phage bactericidal activity in raw milk and referred this to an inhibition of the binding ability of the phage to the host cells in untreated raw milk, which the authors associated with a decrease of phage propagation in raw milk [29,31]. For untreated raw milk, however, previous investigations could show no reduction or merely a reduction in growth compared to an increasing germ density of the controls without phages, but no reduction in germ density compared to the initial values. We wanted to address this concern in the present study by examining the bactericidal activity of a mixture of newly isolated obligately lytic phages against *S. aureus* in milk.

In the present study, the efficacy of obligately lytic phages, specific for *S. aureus*, was investigated against *S. aureus* isolates from mastitis milk samples (host range). Further a phage mixture of three phages was examined in pasteurized and raw milk for its bactericidal activity against *S. aureus*, with regard to a prospective use in the treatment of bovine mastitis. 

## 2. Results

### 2.1. Bacteriophage Host Range: Spot Test and Plaque Assay

**Figure 1:**
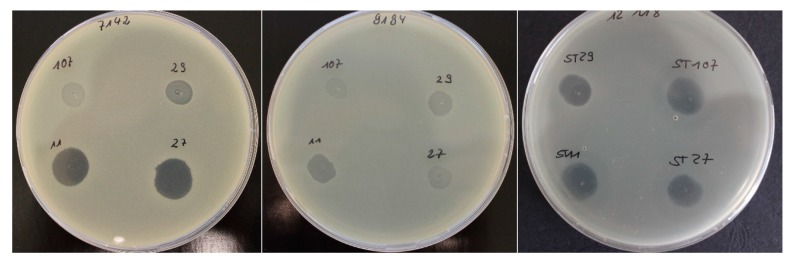
Interpretation of the spot test results.

A spot test and a subsequent plaque assay were performed to determine the lytic efficacy (host range) of three phages (STA1.ST29, EB1.ST11, and EB1.ST27) against 92 *S. aureus* isolates from quarter foremilk samples of subclinical and clinical mastitis cases (Table 1). The subsequent plaque assay was performed for all phage-*S. aureus* combinations with a positive or unclear spot test to exclude false positive results. Figure 1 exemplary shows the interpretation of the spot test results. 

The relative “efficiency of plating” (EOP) was calculated by dividing the phage concentration measured on the test strain by the phage concentration on the respective propagation strain. The results showed both, several isolates with a low relative EOP value, indicating a comparatively low efficiency compared to the propagation strain, and some isolates with a high EOP value, indicating a comparable efficiency of the phage for these isolates (Table 1).

**Figure 2:**
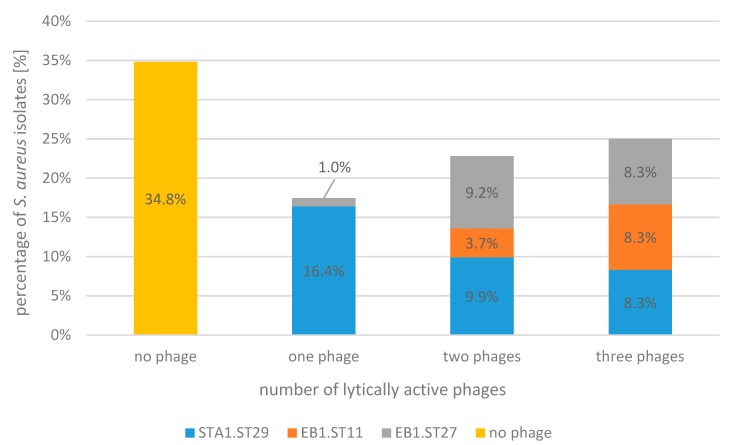
Distribution of the *S. aureus* isolates.

The total of almost two-thirds (65.2%) of the tested isolates was lysed by at least one of the three phages (STA1.ST29, EB1.ST11, and EB1.ST27), while 17.4% of the *S. aureus* isolates were lysed by one phage, 22.8% by two phages and 25% were actually lysed by three phages. The percentage distribution of the individual phages in these categories is shown in Figure 2. A quantity of 34.8% of *S. aureus* isolates could not be lysed by the phages used in the current study.

The single phages, modified by the propagation on different *S. aureus* strains (restriction/modification) [32], showed differences in relation to their individual host range. Phage STA1.ST29 showed a comparatively broad host-range, lysing 63% of the *S. aureus* isolates, followed by phage EB1.ST27 with 44.6% and phage EB1.ST11 with 32.6%. An additional phage, STA1.ST107, was only examined in the spot test. STA1.ST107 showed a host spectrum very similar to EB1.ST11 and EB1.ST27 in the spot test but was excluded from further investigations due to a lack of stability and reproducibility, as the concentration (pfu/mL) of phage STA1.ST107 decreased more than 1-log unit within three months under storage at +6 °C and its propagation resulted in lower concentrations compared to the other phages. 

The above results demonstrated a heterogeneous but broad host range of the individual phages and were used to compose a bacteriophage mixture for further examinations in milk containing the three phages STA1.ST29, EB1.ST11, and EB1.ST27 in equal quantities and with a final concentration of 6.0 × 10^9^ pfu/mL. 

Spot test: 5 µL of each phage solution (1.0 × 10^9^ pfu/mL) were spotted onto the top layer (inoculated with the respective *S. aureus* isolate). Positive result: clear zone greater than or equal to the central area; unclear result: Turbid zone; negative result: no clear or turbid zone, but bacterial lawn.

Spot test results for *S. aureus* isolate 7142 (left) and the four tested phages: STA1.ST107: unclear; STA1.ST29: positive; EB1.ST11: positive; EB1.ST27: positive. Spot test results for *S. aureus* isolate 9194 (middle) and the four tested phages: all unclear. Spot test results for *S. aureus* isolate 12,118 (right) and the four tested phages: all positive. Positive and unclear results were further tested by plaque assay.

The percentage (%) of *S. aureus* isolates (*n* = 92) lysed by none of the phages, one of the three phages (STA1.ST29; EB1.ST11; EB1.ST27), two of the three phages, all three phages, based on the results of the spot test and the plaque assay. The differently colored parts of the bars represent the percentage (%) for the individual phages in each category, respectively.

### 2.2. Phage Bactericidal Activity in Pasteurized Milk

**Figure 3:**
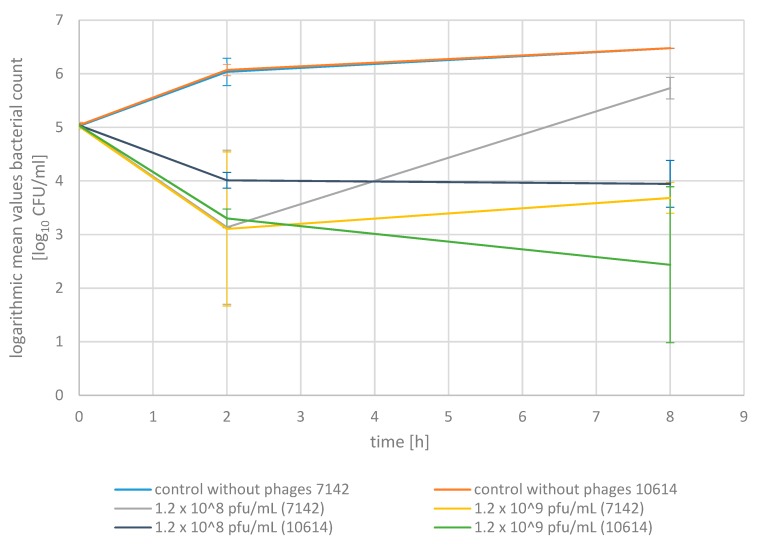
Bactericidal activity of a phage mixture in pasteurized milk with standard deviations. The mean germ density (log_10_ cfu/mL) of the two *S. aureus* isolates 7142 and 10614, respectively, for the control culture without phages (blue: 7142; orange: 10614), the assays with the addition of 0.1 mL phage mixture at a final concentration of 1.2 × 10^8^ pfu/mL (grey: 7142; violet:10614) and 1 mL phage mixture at a final concentration of 1.2 × 10^9^ pfu/mL (yellow: 7142; green: 10614). Inoculation with *S. aureus* at a concentration of averagely 1.1 × 10^5^ cfu/mL. Dilution series were limited at 3.0 × 10^6^ cfu/mL for the investigation in pasteurized milk.

We first investigated the ability of the phage mixture to reduce *S. aureus* in pasteurized milk to gain a stepwise approximation to the in vivo conditions (Figure 3). The pasteurized milk was inoculated with one of the two *S. aureus* isolates (7142; 10614) at 1.1 × 10^5^ cfu/mL, respectively. These two isolates from milk samples were selected according to their lysis by all three phages of the phage mixture. The mean values are presented for each strain. The final phage concentrations added to the *S. aureus* inoculated pasteurized milk were 1.2 × 10^8^ pfu/mL (for the addition of 0.1 mL phage mixture to 5.9 mL inoculated milk) or 1.2 × 10^9^ pfu/mL (for the addition of 1 mL phage mixture to 5 mL inoculated milk), respectively. 

Adding the phage mixture at a volume of 0.1 mL and a final concentration of 1.2 × 10^8^ pfu/mL led to a reduction in the *S. aureus* germ density in pasteurized milk compared to the initial values of 98.9% for *S. aureus* 7142 and 92.1% for *S. aureus* 10,614 after two hours (*p* < 0.05). After eight hours, the reduction was 93.2% for *S. aureus* 10614, but no further reduction could be observed for *S. aureus* strain 7142 as the bacterial density slightly increased in the six-hour interval between the two measurements. However, the germ density remained below the value of the corresponding control cultures without phages after eight hours, with a difference of 84.7% (7142) and 99.8% (10614) (*p* < 0.05). 

Adding the bacteriophage mixture at a volume of 1 mL and a final concentration of 1.2 × 10^9^ pfu/mL resulted in a reduction in bacterial density of 98.8% (7142) and 98.2% (10614) after two hours and of 95.5% (7142) and 99.8% (10614) after 8 h of incubation (*p* < 0.05). Adding the bacteriophages did not lead to an eradication of *S. aureus* at an initial *S. aureus* germ density of averagely 1.1 × 10^5^ cfu/mL. Nevertheless, the *S. aureus* germ density was 99.8% (7142) and 100% (10614) (>3 log units) lower compared to the control cultures without phages after eight hours using 1 mL phage mixture at a final concentration of 1.2 × 10^9^ pfu/mL (*p* < 0.05). 

The addition of the phage mixture at a volume of 1 mL and a concentration of 1.2 × 10^9^ pfu/mL led to a better reduction of the *S. aureus* germ density for an incubation period of 8 h compared to the lower concentration and volume both, with respect to the initial value as well as to the respective value of the control culture.

### 2.3. Phage Bactericidal Activity in Raw Milk

**Figure 4:**
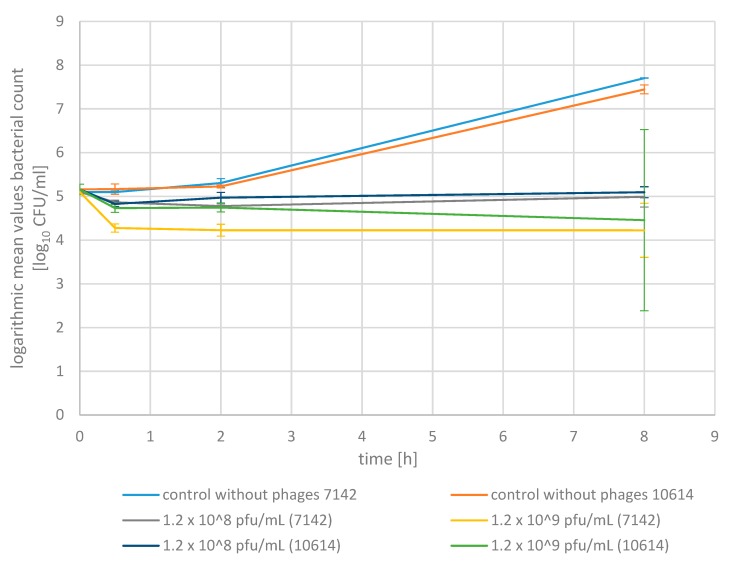
Bactericidal activity of a phage mixture in raw milk with standard deviations. The mean germ density (log_10_ cfu/mL) of the two *S. aureus* isolates 7142 and 10,614, respectively, for the control culture without phages (blue: 7142; orange: 10,614), the assays with the addition of 0.1 mL phage mixture at a final concentration of 1.2 × 10^8^ pfu/mL (grey: 7142; violet:10614) and 1 mL phage mixture at a final concentration of 1.2 × 10^9^ pfu/mL (yellow: 7142; green: 10614). Inoculation with *S. aureus* at a concentration of averagely 1.4 × 10^5^ cfu/mL.

We further tested the efficacy of the bacteriophage mixture to reduce the *S. aureus* isolates in raw milk, as it represents the medium present in the udder (Figure 4). The methods used are the same as for the examination in pasteurized milk. Raw milk was inoculated at an initial value of 1.4 × 10^5^ cfu/mL with one of the two *S. aureus* isolates (7142 and 10614), respectively.

Adding the bacteriophage mixture at a volume of 1 mL and a final concentration of 1.2 × 10^9^ pfu/mL led to an average reduction in *S. aureus* germ density of 84.9% (7142) and 63.1% (10614) within 30 min, 86.6% (7142) and 62.0% (10614) after two hours and 86.6% (7142) and 80.5% (10614) after eight hours of incubation compared to the initial values (*p* < 0.05). When adding a lower volume of 0.1 mL at a ten-fold lower concentration of 1.2 × 10^8^ pfu/mL, a consistently lower reduction in the *S. aureus* germ density of 41.6% (7142) and 54.1% (10614) after 30 min, 52.0% (7142) and 36.3% after two hours and 21.8% (7142) and 14.8% (10614) after eight hours could be observed. Thus, the higher volume (1 mL) and final phage concentration (1.2 × 10^9^ pfu/mL) led to a higher reduction in *S. aureus* germ density in raw milk with a difference of averagely 62.2% after 8 h of incubation compared to the use of the lower volume (0.1 mL) and concentration (1.2 × 10^8^ pfu/mL) (*p* < 0.05).

As described before in pasteurized milk, applying the phage mixture did not lead to an eradication of *S. aureus* from raw milk in the case of the inoculation with an initial concentration of averagely 1.4 × 10^5^ cfu/mL. Nevertheless, the *S. aureus* germ density of samples treated with a volume of 1 mL and a concentration of 1.2 × 10^9^ pfu/mL was 100% (7142) and 99.9% (10614) (>3 log units) lower (*p* < 0.05) and at a lower volume (0.1 mL phage mixture) and a final concentration of 1.2 × 10^8^ pfu/mL, 99.8% (7142) and 99.6% (10614) lower (*p* < 0.05) compared to the control cultures without phages after eight hours of incubation at 37 °C. 

There was also an improvement in reduction ability in raw milk by using a higher volume of phage mixture (1 mL) at a higher final concentration (1.2 × 10^9^ pfu/mL). The phage mixture led to a reduction in germ density with regard to the initial value and the respective values of the control without phages.

## 3. Discussion

Inadequate cure rates for an antimicrobial treatment during lactation as well as resistance to antimicrobial agents in *S. aureus* isolates have been described for *S. aureus* induced mastitis cases [14,16,17]. In order to overcome these issues, the aim of this study was to examine lytic bacteriophages in vitro for their ability to lyse *S. aureus* isolates from mastitis cases, with regard to the future use of phages in mastitis therapy.

In a first step, the host range of the bacteriophages (STA1.ST29; STA1.ST107; EB1.ST11; and EB1.ST27) was determined for a collection of 92 *S. aureus* isolates. The isolates were obtained from quarter foremilk samples of subclinical and clinical mastitis cases and were provided by the Department of Bioprocess Engineering and Microbiology, University of Applied Sciences and Arts Hannover (Germany). Since the isolates originated from 31 different dairy farms in Northern and Eastern Germany, it was assumed that they represented a cross-section of the population of *S. aureus* strains in this area. A spot test was performed in a first step for determining the host range of the phages on the collection of 92 *S. aureus* isolates. Since the clear zone in a spot test is only created by the inhibition of bacterial replication, a plaque assay was used to verify the spot test results by excluding false positive results due to so called “abortive infection”. This term describes the ability of a phage to kill the respective bacterial strain without a productive phage infection [33]. This extended investigation of lytic phage activity on a large *S. aureus* collection (*n* = 92) from mastitis milk samples differentiates the present study design from previous studies using smaller strain collections or merely performing a spot test for determining the host range and it is to our knowledge the first investigating phages against *S. aureus* isolates from German dairy herds.

The determination of the host range in the present study showed that almost two-thirds (65.2%) of the 92 *S. aureus* isolates could be lysed by at least one of the tested phages, while 47.8% were actually lysed by two or more of the phages under investigation. These results led to the preparation of a bacteriophage mixture to increase the host range and to minimize the potential risk of developing resistance to individual phages [24,27]. It has already been described that a bacteriophage mixture is significantly more efficient in lysing *S. aureus* isolates than a single phage [31]. 

The relative EOP is described in the literature as the concentration of the individual phage for the tested bacterial strain compared to the maximum phage concentration measured by plaque assay under different conditions [34]. In the case of our study, the maximum concentration to be achieved refers to the highest phage concentration measured for its propagation strain (ST11; ST27 or ST29). The relative “efficiency of plating” varies according to the different test isolates examined in the present study. There are some isolates with a comparatively high EOP value, which indicates an efficiency in phage replication comparable to that on the propagation strain. Several isolates have a low EOP value, which suggests that phage in situ replication is not as efficient or at least not as fast for a particular targeted strain other than the propagation strain. 

The differences in the EOP of a phage in relation to the different test isolates are explained in the literature by the fact that the EOP depends on various factors such as the agar concentration in the top agar or the *S. aureus* germ density [34]. However, these factors were constant in our investigations and therefore had no influence on the different EOP values. In addition, the influence of specific host factors (masking by O-antigens; presence of restriction endonucleases) on the EOP is described and accordingly a difference of up to a factor of ten between the individual test isolates is not uncommon [34]. Further, a comparatively low ability to induce infection, possibly due to reduced specific structures on the host cell surface to which the phages bind, may increase the probability of delayed infection initiation [35]. 

A possible reason for the non-sensitivity of isolates (34.8%) might be the fact that the *S. aureus* phages in the current study belong to the families of Myo- (STA1) and Podoviridae (EB1). Thus, they represent only two of the three common *S. aureus*-bacteriophage families. This leads to the assumption that the isolation and additional application of a lytic phage from the third family (Siphoviridae) might result in an increased host range of a phage mixture. 

All currently known staphylococcal phages require wall teichoic acid (WTA) for adsorption and infection [36,37]. WTA is an anionic glycopolymer that is linked to the peptidoglycan layer of most gram-positive bacteria [38]. The backbone of the WTA of most *S. aureus* strains contains 40–60 phosphodiester-linked polyribitol phosphate units, further decorated with three substitutions, D-alanine, α-GlcNAc or β-GlcNAc [39]. While Myoviridae, like phage K, bind both to the WTA backbone and to α-GlcNAc-modified WTA and thus have the broadest host range [36,39], Siphoviridae, like phi77, depend on GlcNAc-modified WTA and Podoviridae, like PS66, specifically depend on β-GlcNAc-modified WTA [37]. An optimal phage cocktail to fight *S. aureus* would thus include bacteriophages of all three families, with the idea that point mutations that eliminate the receptor of one bacteriophage might not automatically also lead to the exclusion of the other bacteriophages in the cocktail. However, a *tag0* mutant as described in [36], entirely devoid of WTA, would be resistant against all bacteriophages of such an optimal cocktail. However, this might be of minor importance during practical applications. First, mutants devoid of WTA show a significant growth defect compared to wildtype strains and second, such mutants would be sensitive to ß-lactam antibiotics, as the MRSA resistance phenotype in *S. aureus* also depends on the presence of glycosylated WTA [40]. Thus, a combination of phage therapy and antibiotic treatment might lead to synergistic effects and would reduce the selection of both phage resistant and antibiotic resistant variants of *S. aureus*. 

A direct isolation of lytic *S. aureus*-specific Siphoviridae from milk or sewage water generally proves difficult. Nonetheless, Garcia et al. [41] obtained two lytic phages (Siphoviridae) from their milk isolated temperate counterparts by DNA random deletion. O’Flaherty et al. [42] actually isolated two lytic Siphoviridae from farmyard slurry. Both achieved good results against *S. aureus* isolates [41,42].

Furthermore, we investigated the bactericidal activity of a phage mixture in pasteurized and raw milk for an approximation to the conditions prevailing in vivo. Milk represents a complex medium of polydisperse character composed of lipids, milk proteins (caseins, whey proteins), enzymes, lactose, minerals, trace elements, and vitamins [28]. Therefore, it was necessary to examine the lytic activity of the phages in bovine milk with regard to a future intramammary application in mastitis therapy. The two *S. aureus* isolates used for inoculating pasteurized and raw milk were selected based on their sensitivity to all three phages within the mixture, as determined in the first set of experiments. They represented only a small part of the total *S. aureus* spectrum tested, but were assumed to provide important information on the antibacterial potential of the bacteriophage mixture in milk. The bacteriophage mixture contained the phages STA1.ST29, EB1.ST11, and EB1.ST27 in equal quantities and with a concentration of 6.0 × 10^9^ pfu/mL. We investigated the influence of the phage mixture added in different volumes (0.1 mL; 1 mL) leading to different final concentrations (1.2 × 10^8^ pfu/mL; 1.2 × 10^9^ pfu/mL) and the influence of the incubation time on the reduction of the *S. aureus* germ density (cfu/mL) in milk. The trials were conducted with a view to an in vivo use and an application interval of eight hours (intermediate milking time), considered most practicable for treatment during lactation. 

While the control without phages showed a steady increase of the bacterial count, adding the phage mixture at 1 mL and 1.2 × 10^9^ pfu/mL led to a significant reduction of the *S. aureus* germ density, both with respect to the control without phages as well as to the initial value, which has to be emphasized.

In pasteurized milk, an adequate reduction in the *S. aureus* germ density for an eight-hour incubation period (37 °C) was only achieved when a higher volume and concentration of bacteriophages (1 mL; 1.2 × 10^9^ pfu/mL) was used, with a reduction of averagely 97.7% after eight hours, compared to the initial germ density (*p* < 0.05). Compared to the values of the control culture at t_8h_, a reduction of up to 100% (>3 log units) was achieved after eight hours of incubation (*p* < 0.05). 

Garcia et al. [31] achieved a similar reduction of 3.6 log units compared to the values of the control culture in pasteurized milk by using a phage mixture of two temperate phages after ten hours of incubation, but no significant reduction compared to the initial values. In the current study we used a comparably small number of three obligate lytic phages for the phage mixture, which exclusively enter the lytic propagation cycle, commonly lead to a faster lysis and should be preferred for a therapeutic use [25,27]. 

In raw milk, adding a higher volume and concentration of phage mixture (1 mL; 1.2 × 10^9^ pfu/mL), led to a higher reduction with a difference of 62.2% after 8 h compared to the use of a lower volume at a ten-fold lower concentration (0.1 mL; 1.2 × 10^8^ pfu/mL) (*p* < 0.05). The incubation period of 8 h led to a steady increase in the reduction of the *S. aureus* germ density for the use of a higher volume and concentration (1 mL; 1.2 × 10^9^ pfu/mL) with a difference of 10% compared to the reduction after 30 min (*p* < 0.05). In contrast, a decrease in the reduction ability over time for the lower volume and concentration (0.1 mL; 1.2 × 10^8^ pfu/mL) with a difference of 28.6% compared to the reduction of bacterial density after 30 min could be observed (*p* < 0.05). This shows that only the higher phage concentration was sufficient to adequately reduce the *S. aureus* germ density for an incubation period of 8 h in raw milk. 

Adding a phage mixture in the present study with a volume of 1 mL at a concentration of 1.2 × 10^9^ pfu/mL to *S. aureus*-inoculated raw milk resulted in a reduction of averagely 83.6% after eight hours compared to the initial value, and to a reduction of averagely 100% after 8 hours compared to the control cultures at the same time. When considering these results, it has to be noted that the ability of the phage mixture to reduce the *S. aureus* germ density is maintained in raw milk with only a moderate decrease compared to pasteurized milk, with an initial *S. aureus*-inoculation of 1 × 10^5^ cfu/mL.

In contrast, other studies on the use of bacteriophages against *S. aureus* showed considerably lower or no reduction rates in raw milk and assumed that the ingredients interfere with the ability of the phages to bind to their host cells. In two different studies on the bactericidal activity of a single application of phage K, an inhibition of phage binding to its host cells in raw milk and whey was described [29,30]. Phage K is an exclusively lytic *S. aureus* specific phage with a broad host range belonging to the *Myoviridae* family [43], and is closely related to the STA1 bacteriophage studied here. Immunoglobulin activity, the clumping of *S. aureus* cells associated with fat globules, or whey proteins are suspected to be possible causes limiting phage K adsorption to the host cell surface in raw milk [29,30]. 

The design of the previous publications shows some divergences in comparison to the current study. In particular, only a solitary phage K was used in contrast to the application of a bacteriophage mixture as used in the present study. The combined use of several lytic phages offers advantages regarding the host range and therefore increases the bactericidal activity [31].

Regarding the application of a combined use of phages in the medium milk, a mixture of two temperate phages (10^4^ to 10^5^ pfu/mL) against *S. aureus* (10^2^ cfu/mL) has been examined in a previous study [31]. These investigations were carried out in UHT milk, pasteurized milk and in semi-skimmed and unskimmed raw milk. The authors described significantly lower *S. aureus* numbers for the use of this mixture of temperate phages in raw milk after an incubation period of 11 h compared to the control without phages. Nevertheless, they found a considerable decline in the ability of the phages to reduce *S. aureus* in raw milk compared to the results obtained in pasteurized milk.

In contrast, the results of the present study show not only a relative reduction compared to the control without phages, but also an absolute reduction compared to the initial bacterial density; with only a slight decrease in bactericidal activity of phages (14.1% for absolute reduction) in raw milk compared to the results in pasteurized milk.

The use of higher initial concentrations of phages and *S. aureus* represent differences from the previous study. At the same time, the use of newly isolated obligate lytic phages could be a possible reason for the differences from the results of the previous study and thus represents an important factor with regard to the bactericidal activity of the bacteriophages used. Since obligate lytic phages generally lead to a faster lysis of the bacterium and have a lower lysogenic and transduction potential, this application offers a decisive advantage for an application in phage therapy. In further in vivo studies, a phage concentration as used in this study should also be applied.

In the present study, the continuous propagation of initially two genetically different phages (STA1 and EB1) on four different *S. aureus* strains (two isolated from mastitis samples) has resulted in four phages with different host ranges. This leads to the hypothesis that using *S. aureus* isolates from mastitis cases (ST 11 and ST 27) for the propagation of the phages (EB1.ST11 and EB1.ST27) in this study might be another reason for the bactericidal activity of the bacteriophage mixture in raw milk.

Utilizing a bacteriophage mixture in pasteurized milk and in raw milk in the present study, with an initial germ density of ~1.0 × 10^5^ cfu/mL, did not lead to a complete eradication of *S. aureus*. This observation is comparable to the results of another publication [31]. Garcia et al. [31] suggested that a total reduction in *S. aureus* in milk could not be achieved due to a low phage-cell ratio at the end of their investigation. In the same way, the stationary phase in the growth of *S. aureus* could also be responsible for the incomplete reduction, as the authors assume in their paper. Accordingly, low *S. aureus* cell densities and a stationary phase of the bacterial culture at the end of the incubation period could also have been the limiting factor in the present study. At the same time, this phage property also includes the advantage of so-called “self-limitation”, which is considered beneficial for a therapeutic approach by other authors [22].

However, the results of the present study already demonstrated that using the phage mixture at a higher volume of 1 mL and a higher final concentration of 1.2 × 10^9^ pfu/mL led to a higher reduction in *S. aureus* germ density in raw milk in vitro (*p* < 0.05). It is conceivable that the higher used volume led to a better distribution in vitro and consequently to an improved ability of the phages to target and bind to their host cells [44]. Further investigations on this issue should be conducted in vivo to determine a volume that results in an adequate dispersion in the udder. In order to adapt the application frequencies in vivo to an intermediate milking time of 8 h, our findings suggest that for field trials a calculation based on a concentration of 1.2 × 10^9^ pfu/mL would be necessary to achieve an appropriate bactericidal activity at a bacterial density of 1 × 10^5^ cfu/mL. 

The shedding of *S. aureus* from infected mammary gland quarters varies depending on whether a subclinical or clinical infection is present and may increase after a stress event and shedding of 10^5^ cfu/mL is not uncommon. The examined shedding of *S. aureus* in quarter-foremilk samples from subclinically infected mammary gland quarters after a stress event had a median value of 88 cfu/mL [45]. Only as an example, calculated for a subclinical infected udder quarter with an average *S. aureus* germ density of 100 cfu/mL, a minimum phage concentration of 1 × 10^6^ pfu/mL would be required. Assuming an increase in the milk volume between milking times to a total of 5 L/quarter of udder, an intra-mammary application every 8 h would require the injection of 5.0 × 10^9^ pfu/mL. If a volume of 10 mL phage solution is administered, it should have the concentration of 5.0 × 10^8^ pfu/mL. 

Further investigations in vivo are necessary, as the conditions cannot be adequately reproduced in vitro due to the complex structure and functional characteristics of the bovine udder (immune response, dispersion, pharmacokinetic). They should first include a tolerability assessment for administering a phage mixture in the teat sinus of clinical healthy cows. Further investigations should be conducted to determine an adequate concentration and volume of phage solution for an intramammary administration. This should be followed by an examination of the bacteriological cure rates in comparison to an intramammary treatment with antimicrobial agents during lactation. Based on these in vivo examinations, an evaluation of the applicability and the economics of the phage mixture for mastitis therapy has to be performed.

Other studies, which investigated the use of phages in animal experiments already showed promising results [22,46,47]. Capparelli et al. [23] showed the activity of *S. aureus* bacteriophages against local and systemic *S. aureus* infections as well as intracellular and methicillin resistant strains of *S. aureus* (MRSA) in mice. Phage-treated mice neither showed a stimulated production of neutralizing antibodies nor did they suffer any side effects. These in vivo examinations point out that phage therapy in animals is able to generate good cure rates without adversely affecting animal health. Further studies examined the use of the single phage ΦSA012 or a mixture of two phages (Myo- and Podoviridae) isolated from sewage water against *S. aureus* isolated from bovine mastitis milk in a mouse model [46,47]. The authors reported reduced *S. aureus* proliferation and a decrease of the inflammatory response in the mouse mammals, which represents an important result with regard to the therapy of bovine intramammary infections (IMIs) using phages. However, the authors argue that a direct comparison between mouse mammals and bovine udders is not possible since their anatomy and physiology differ. The authors therefore indicate further investigations in bovine mammary glands [46,47]. In addition, there is the fact that the quantities of milk ingredients differ between animal species [48]. The investigation of the bactericidal activity of phages in raw milk from cattle, as described in the present study, is therefore an essential basis concerning the future use in the therapy of bovine mastitis, as it represents the medium in the bovine udder.

The number of in vivo studies on the use of phages for *S. aureus* mastitis treatment in cattle is quite low [42,49]. O’Flaherty et al. [42] administered three *S. aureus* phages intramammarily for tolerability assessment and found no increased somatic cell count (SCC) and thus no increased immune response to a relatively high phage concentration of 10^8^ pfu/mL. Furthermore, they proposed the use of phages in special formulations as teat-dips or teat-washes, as they had already successfully examined phages in the form of hand-wash solutions against *S. aureus* in hospitals in a previous study [50]. This underlines the additional possibility of using phages prophylactically in the case of bacterial colonization of the bovine udder and udder skin. Gill et al. [49] investigated the single use of bacteriophage K for treating subclinical *S. aureus* mastitis in the lactation period. A cure rate of three of 18 quarters (16.7%) was achieved, whereas in the control group, zero of 20 quarters were cured. However, these results were not statistically significant. Intramammary infusion of the phage into quarters already infected with *S. aureus* had in contrast to the application in healthy quarters no adverse effects, as no increase in the somatic cell count was observed. 

The phage mixture of the present study led to a considerable reduction of the *S. aureus* germ density in raw milk, which might lead to improved results in vivo compared to the previous examinations. In their study, Gill et al. [49] administered an amount of 10 mL intramammary infusion of phage K (1.25 × 10^11^ pfu/mL) once per day for five days. In relation to the findings of our study, it would be worth considering administering a combination of several obligately lytic phages in form of a phage mixture to increase the host range and to fulfill safety requirements in phage therapy [25,27,31]. At the same time, the exemplary calculation based on our results shows that even a lower concentration (5 × 10^8^ pfu/mL) at a comparably high volume (10 mL) might be sufficient for the in vivo application of a phage mixture. A practicable eight-hour application interval (intermediate milking period) could be used in further in vivo trials. 

The results of the present examinations indicate that bacteriophage therapy could be a promising measure in mastitis treatment. Indeed, it might be a future alternative in order to improve the cure rates of *S. aureus* mastitis during lactation, to reduce the number of antimicrobial agents and to counteract the development of antimicrobial resistance in *S. aureus*. Further investigations need to focus to a greater extent on studying obligately lytic phage mixtures in vivo for *S. aureus* mastitis therapy. The results of the present study offer an incentive to do so, as the use of a lytic bacteriophage mixture in raw milk led to significant and promising results against *S. aureus* isolates of mastitis cases.

## 4. Materials and Methods 

### 4.1. S. aureus Strains and Culture Methods

The *S. aureus* isolates (*n* = 92) for the host range determination of phages were provided by the Department of Bioprocess Engineering and Microbiology, University of Applied Sciences and Arts Hannover (Germany). The isolates were obtained from quarter foremilk samples of clinical and subclinical bovine mastitis cases collected on 31 different farms in North and East Germany in order to obtain an adequate reproduction of the strain variety in the field. All *S. aureus* isolates were screened nuc gene positive. The isolates were stored at −80 °C, after a 24 h-culture of the respective strain in brain heart broth, with the addition of 20% glycerine, and, before use, incubated on esculin blood agar (Oxoid Deutschland GmbH, Wesel, Germany) for 24 h at 37 °C. Directly before every trial, an overnight culture of the respective strain was incubated in Luria-Bertani (LB) broth (trypton/pepton from casein: 1 g/100 mL; yeast extract, micro-granulated: 0.5 g/100 mL; sodium chloride (NaCl): 0.5 g/100 mL; Carl Roth GmbH & Co. KG, Karlsruhe, Germany) at 37 °C. LB broth was used for bacterial growth in suspension, phage spot test and double-layer agar (DLA) technique. Broth was supplemented with agar (Agar Agar; Carl Roth GmbH & Co. KG, Karlsruhe, Germany) at concentrations of 1.5% for bottom and 0.5% for top agar. Sterile dextrose was added to the top agar at 1.1% before use.

The *S. aureus* isolates ST11, ST27, ST29, and ST107, isolated and provided by PTC GmbH, Bönen, Germany, were used for routine phage propagation (Table 2). Phage propagation strains ST11 and ST27 originated from mastitis milk samples.

### 4.2. Bacteriophages 

Bacteriophages EB1.ST11, EB1.ST27, STA1.ST29, and STA1.ST107, isolated and provided by the PTC GmbH, Bönen, Germany, are obligately lytic phages belonging to the order of *Caudovirales* and represent the families of *Myoviridae* (STA1) and *Podoviridae* (EB1), as their genome sequence analyses showed.

**Figure 5:**
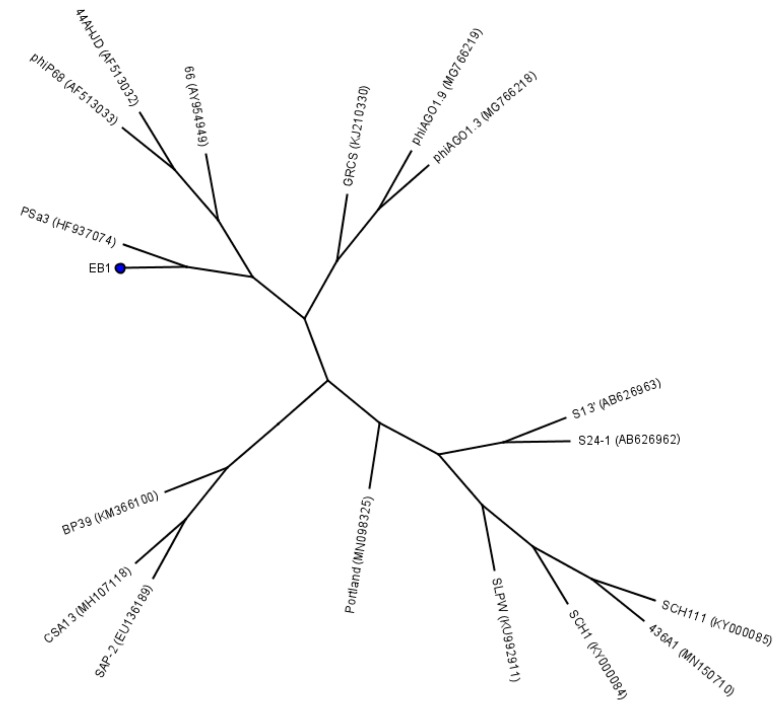
UPGMA tree constructed by the Geneious tree builder (software version 4.8) on the basis of a multiple genome alignment of 18 *S. aureus* phages belonging to the *Podoviridae* family. The position of the phage EB1 is marked by a dot.

**Figure 6:**
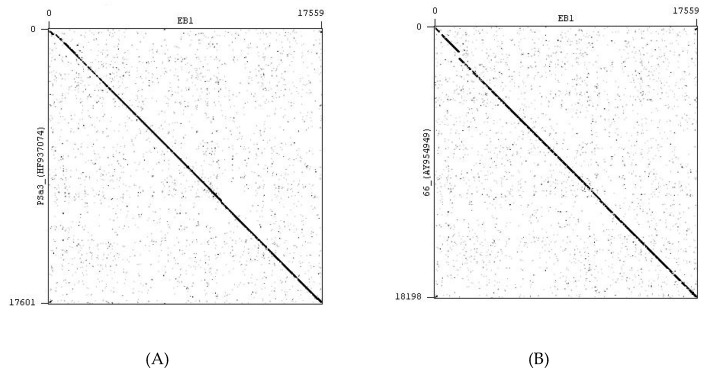

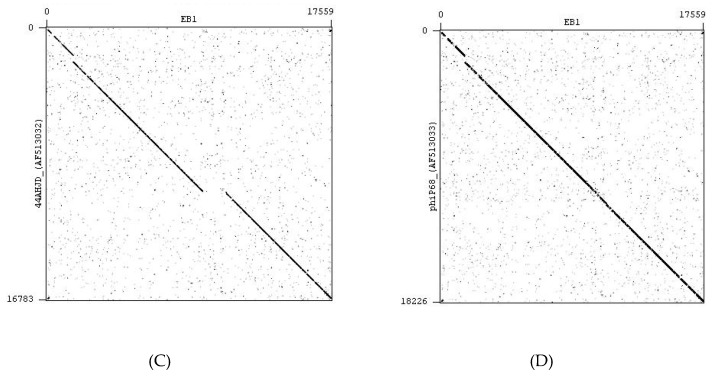
Local comparison of two phage genomic sequences (dotplot) calculated by Gepard software version 1.40: **A**—comparison of genomic sequences of phages EB1 and PSa3; **B**—comparison of genomic sequences of phages EB1 and 66; **C**—comparison of genomic sequences of phages EB1 and 44AHDJ; **D**—comparison of genomic sequences of phages EB1 and phiP68.

Phage EB1 was isolated from pig manure (Table 2) and is closely related to several members of the genus *Rosenblumvirus*, namely, PSa3 (93.5% genome identity), which was isolated and characterized by Kraushaar et al. [51], phiP68 (89.3% genome identity) and 44AHJD (82.5% genome identity) from the collection of the Felix d’Herelle Reference Center for Bacterial Viruses, Quebec, Canada [52], as well as phage 66 from the National Collection of Type Cultures (London) [53]. The genomic similarity between EB1 and other known bacteriophages of *Rosenblumvirus* is visually demonstrated by a tree, which was constructed with the help of the Geneious (version 4.8) tree builder using the UPGMA tree build method and the HKY Genetic Distance Model (Figure 5). The local genomic similarities between EB1 and a group of phiP68-like phages are illustrated as dotplots (Figure 6), which were created by Gepard version 1.40 [54].

**Figure 7:**
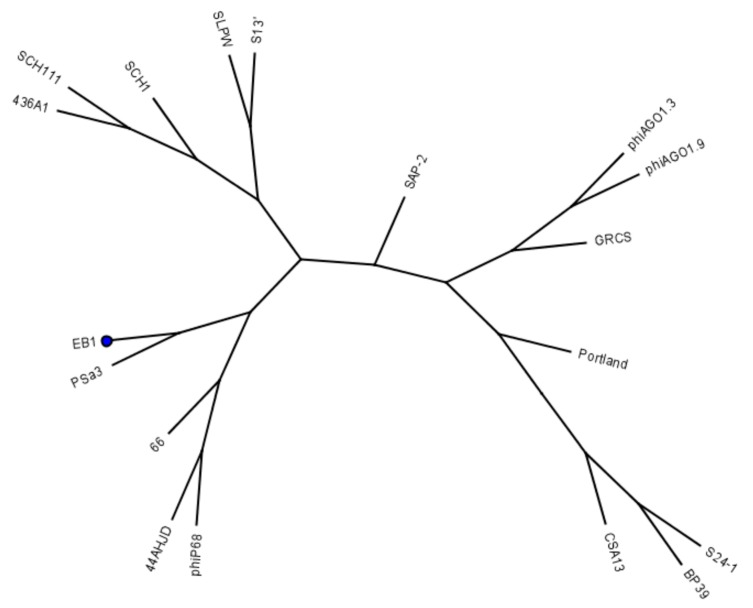
UPGMA tree constructed by the Geneious tree builder (software verion 4.8) on the basis of a multiple protein alignment of 18 receptor binding proteins (homologs of phiP68-encoded ORF17) present in the genomes of *S. aureus* phages belonging to the *Podoviridae* family. The position of the receptor binding protein encoded by the phage EB1 is marked by a dot.

The phages phiP68, 44AHDJ and 66 are known to adsorb to *S. aureus* wall teichoic acid (WTA) [38] modified with β-GlcNAc [37]. The phiP68-encoded ORF17 was found to be a virion-associated muralytic enzyme [55] and a receptor binding protein [56]. EB1 encodes a protein, showing 96.5% identity to the phiP68-encoded ORF17. The 648 amino acids long protein has only 23 amino acid substitutions from its phiP68 counterpart and the majority of them represent changes for structurally similar amino acids. Homologs of ORF17, encoded by other phiP68-like bacteriophages, demonstrate a close evolutionary relationship, forming a separate group within the phylogenetic tree (Figure 7). 

Phage STA1 was isolated in a wastewater facility and is closely related to phage K (89.1% genome identity) [43] and other members of the genus *Kayvirus*.

The two genetically distinct phages were grown on different host strains, which leads to epigenetic modifications that may result in a different host range [32]. All bacteriophages used in this study are available upon request from the PTC GmbH, Bönen, Germany. 

### 4.3. Bacteriophage Propagation 

Phages were routinely propagated by inoculating LB-broth with 1% of an overnight culture of the respective *S. aureus* propagation strain (either ST11; ST27; ST29; or ST107). The initial value of the optical density at 600 nm (OD_600_) was determined by a photometer (SPEKOL^®^1500, Analytik Jena AG, Jena, Germany). Data were collected every 30 min until an OD_600_ of 0.3 was reached. To the exponentially growing *S. aureus* cells, 1% of a phage stock solution with a concentration >1.5 × 10^10^ pfu/mL (EB1.ST11), >1.3 × 10^9^ pfu/mL (EB1.ST27), >1.6 × 10^9^ pfu/mL (STA1.ST29), >2.0 × 10^9^ pfu/mL (STA1.ST107) was added. The OD_600_ was measured further at half-hourly intervals. Chloroform was added at 1 ‰ (one per mil) when a decrease in the OD_600_ was observed and the solution was then centrifuged at 10,000× *g* for 20 min at room temperature. The supernatant was removed and sterile filtered (0.45 µm-pore-size filter, Minisart^®^NML Plus/NY Plus; Sartorius AG, Göttingen, Germany) before phage plaque formation (pfu/mL) was determined by using a double-layer-agar-technique, as described below. The sterile filtered phage solution was stored at +6 °C in the dark until further use and the phage concentration (pfu/mL) was routinely determined.

### 4.4. Spot Test

A spot test was used to investigate the lytic activity of the phages EB1.ST11, EB1.ST27, STA1.ST29, and STA1.ST107 against a collection of 92 *S. aureus* isolates from mastitis cases to determine their host range. Briefly, 100 µL of a bacterial overnight culture were added to 5 mL of top agar (0.5% agar), supplemented with sterile dextrose before use. The solution was mixed by a Vortex Mixer (Vortex Genie^®^2; Scientific Industries Inc., Bohemia, NY, USA) and poured onto a petri dish (Ø 94 × 16 mm) prepared with 10 mL of LB-bottom agar. The petri dish was gently swirled and dried at room temperature before 5 µL of a phage solution (1.0 × 10^9^ pfu/mL) were spotted onto the top layer. The plates were allowed to dry at room temperature before being incubated inverted at 37 °C for 18 h. The spot test was considered positive (+) if a clear zone appeared at the site of application, and considered negative (−) if no clear zone appeared. If only a turbid spot could be seen, it was defined as an unclear result (+/−) (Table 1). In cases of positive (+) and unclear (+/−) spot test results, a plaque assay was performed as a second step to verify lytic phage activity for the respective *S. aureus* isolates.

### 4.5. Plaque Assay

Spot test results were verified by a plaque assay, using the double-layer-agar-technique, modified to the one described by Sambrook and Russel (Table 2) [57]. This method was used to exclude false positive spot test results [33,34]. Briefly, 100 µL of a serial dilution of phage solution (Ringer’s solution; Merck KGaA, Darmstadt, Germany) were added to 100 µL of a bacterial overnight culture of the respective *S. aureus* strain. After incubation for 5 min at room temperature, 5 mL of top agar (0.5% agar) were added, mixed by a Vortex Mixer and poured onto a petri dish (Ø 94 × 16 mm) prepared with 10 mL of LB-bottom agar. The petri dish was gently swirled and dried at room temperature for 30 min. Inverted plates were incubated at 37 °C for 18 h. Plaque forming units per milliliter (pfu/mL) were determined and the relative “efficiency of plating” (EOP) was calculated by dividing the phage concentration (pfu/mL) for the test strain by the phage concentration for the respective propagation strain (ST29; ST11; or ST27) [34]. Thus, the spot test results were confirmed by the active formation of a plaque by the phage in a lawn of the target bacterial strain.

A bacteriophage mixture was prepared to investigate the potential of a combined use of different phages against *S. aureus* in milk. The phage mixture was prepared of the three phages EB1.ST11, EB1.ST27, and STA1.ST29. These phages were selected for their host range determined by spot test and plaque assay, for a high concentration of phages after propagation (>1.0 × 10^9^ pfu/mL) and for an adequate storage stability at +6 °C. The latter was defined in terms of a decrease in concentration (pfu/mL) less than one log unit over a period of three months.

### 4.6. Phage Bactericidal Activity in Pasteurized Milk

The bactericidal activity of the phage mixture against *S. aureus* isolates from mastitis cases was investigated in milk in order to achieve a gradual adaptation to the conditions in vivo. The two *S. aureus* isolates used for inoculation originated from different dairy farms and had shown good results in the previous assays with regard to their sensitivity towards all three phages in the mixture.

Pasteurized milk was obtained by heating raw milk (<20,000 SCC/mL and quarter) to 63 °C for 30 min. The raw milk had been tested previously for the absence of *S. aureus* and antibiotic residues by direct plating and a Brilliant Black Reduction Test (Delvotest^®^BR Brilliant, DSM; MILKU Tierhygiene GmbH, Bovenden, Germany). The pasteurized milk was inoculated with *S. aureus* isolates 7142 or 10,614 at concentrations of 1.1 × 10^5^ cfu/mL, respectively. 

We added either 0.1 mL phage mixture to 5.9 mL inoculated milk or 1 mL phage mixture to 5 mL inoculated milk. The final phage concentrations were 1.2 × 10^8^ pfu/mL or 1.2 × 10^9^ pfu/mL for the different added volumes. Assays were mixed by a Vortex Mixer before incubating at 37 °C without shaking and samples were taken at the beginning of the trial (t_0h_), after two hours (t_2h_) and after eight hours (t_8h_). The two-hour incubation time was related to the average replication time of the phages determined in the propagation trial, whereas the eight-hour incubation time referred to an intermediate milking period at milking-frequency of three times per day. It was used to indicate whether administering a phage mixture only once per milking time (eight hours) would lead to an adequate reduction. For examining the *S. aureus* germ density (cfu/mL), diluted suspensions (Ringer’s solution; Merck KGaA, Darmstadt, Germany) were plated by the spatula method with 100 µL sample material [58]. Briefly, this was carried out by plating serial dilutions (Ringer’s solution, Merck KGaA, Darmstadt, Germany) and subsequent calculation of colony forming units per milliliter (cfu/mL) after incubation at 37 °C for 24 h. Five measurements were collected for each data point. Pasteurized milk inoculated with *S. aureus* (7142 or 10614) was used as control culture without phages, *S. aureus*-free pasteurized milk as negative control.

### 4.7. Phage Bactericidal Activity in Raw Milk

The investigation of the phage bactericidal activity in raw milk was carried out as described above in pasteurized milk and was used to obtain a further adaption to in vivo conditions. Raw milk was tested previously for the absence of *S. aureus* and antibiotic residues. Raw milk was inoculated with the *S. aureus* isolates 7142 or 10614 at concentrations of 1.4 × 10^5^ cfu/mL. Furthermore, we added either 0.1 mL phage mixture to 5.9 mL inoculated milk or 1 mL phage mixture to 5 mL inoculated milk, with a final phage concentration of 1.2 × 10^8^ pfu/mL or 1.2 × 10^9^ pfu/mL, respectively. The preparations were mixed by a Vortex Mixer before incubating at 37 °C without shaking and examined for their *S. aureus* germ density at the beginning (t_0h_), after 30 min (t_30min_), after two hours (t_2h_) and after eight hours (t_8h_) by the spatula method with 100 µL sample material [58]. Trials were carried out in quintuplicate, including control cultures without phages (*S. aureus* inoculated raw milk) and negative control (non-inoculated raw milk).

### 4.8. Statistical Analysis

To obtain statistically relevant data for the examinations in milk, five measurements were collected for each data point. A statistical calculation was performed to investigate the influence of the different concentrations, achieved by different added volumes, and the incubation time on the *S. aureus* germ density. Logarithmic transformations of cfu (log10) were applied to approximate normal distribution and were analyzed by ANOVA using SPSS 25.0 (IBM SPSS 25.0.0.0., Armonk, USA). P values <0.05 were considered significant.

## 5. Conclusions

We determined the host range of obligately lytic bacteriophages against *S. aureus* isolates from clinical and subclinical mastitis cases. The examinations identified bacteriophages with a broad host range. In a second step, we examined the bactericidal activity of a three-component bacteriophage mixture for the reduction of *S. aureus* in pasteurized and raw milk. The results showed a significant reduction of the *S. aureus* germ density in pasteurized milk and a slightly lower but still significant reduction in raw milk after eight hours of incubation at 37 °C. The reduction in bacterial density was shown both in comparison to the initial values and to the values of the controls without phages. The results are promising and support further investigations into the use of bacteriophages in the treatment of bovine *S. aureus* mastitis.

## Figures and Tables

**Table 1:** Host range of the phages: Results of a spot test and a plaque assay (relative efficiency of plating (EOP)).

	Spot test/relative EOP					
	STA1.ST29		EB1.ST11		EB1.ST27	
*S. aureus* isolates	spot test ^a^	EOP ^b,c^	spot test ^a^	EOP ^b,d^	spot test ^a^	EOP ^b,e^
5208	+/−		+	6.67 × 10^−5^	+	4.93 × 10^−5^
5209	+/−		+	1.20 × 10^−4^	+	8.42 × 10^−6^
5210	+	3.88 × 10^−5^	+	5.70 × 10^−4^	+/−	
5213	+/−		+/−		−	
5214	+	6.95 × 10^−2^	+	1.15 × 10^−7^	+	1.76 × 10^−5^
5219	+/−		−		−	
5220	+/−		−		−	
5221	+/−		+/−		−	
5222	+/−		+/−		−	
5223	+/−		+/−		−	
5224	+/−		+/−		−	
5225	+/−		+/−		−	
5226	+/−		+/−		−	
5228	+/−		+/−		−	
5233	+/−		+/−		−	
5235	+/−		+/−		−	
7135	+	9.07 × 10^−3^	+	5.97 × 10^−9^	+	1.24 × 10^−6^
**7142**	+	1.23 × 10^−2^	+	7.13 × 10^−4^	+	1.79 × 10^−2^
8046	+	4.05 × 10^−1^	+	2.74 × 10^−6^	+	5.86 × 10^−5^
9184	+	5.20 × 10^−1^	+	1.58 × 10^−6^	+	7.57 × 10^−5^
9185	+/−		+/−		−	
9192	+/−		+/−		−	
9194	+/−		+/−		+/−	
9210	+/−		+	2.32 × 10^−4^	+	2.74 × 10^−4^
9220	+/−		+/−		+/−	
9226	+/−		+/−		−	
9234	+/−		+/−		−	
9240	+/−		+/−		−	
9242	+/−		+/−		−	
9264	+	1.95 × 10^−1^	+	2.53 × 10^−4^	+	4.70 × 10^−5^
9267	+	1.81 × 10^−1^	+/−		+	5.59 × 10^−6^
9271	+/−		+/−		−	
9276	+/−		+/−		−	
9833	+	1.12 × 10^−1^	+/−		+	9.87 × 10^−8^
9854	+	9.51 × 10^−3^	+/−		+/−	
9858	+	1.43 × 10^0^	+/−		+	6.58 × 10^−7^
9899	+	1.55 × 10^−2^	+/−		+/−	
9931	+	3.05 × 10^−2^	+/−		+/−	
9996	+	1.33 × 10^−2^	+/−		+	3.29 × 10^−7^
9999	+	6.90 × 10^−2^	+	9.18 × 10^−8^	+	1.72 × 10^−6^
10048	+	1.16 × 10^−2^	+/−		+/−	
10141	+	1.34 × 10^−2^	+/−		+	3.29 × 10^−7^
10154	+	1.32 × 10^−2^	+/−		+	6.58 × 10^−7^
10170	+	3.78 × 10^−3^	+	1.05 × 10^−7^	+	5.26 × 10^−6^
10172	+/−		−		−	
10201	+	9.76 × 10^−3^	+/−		+	3.29 × 10^−7^
10237	+	1.57 × 10^−2^	+/−		+/−	
10366	+	1.23 × 10^−2^	+/−		+/−	
10451	+/−		−		−	
10455	+	2.93 × 10^−4^	+/−		−	
10490	+	5.44 × 10^−4^	−		−	
10538	+	4.39 × 10^−3^	+/−		+/−	
10574	+	1.03 × 10^−2^	−		+	2.39 × 10^−5^
**10614**	+	3.93 × 10^−2^	+	1.34 × 10^−3^	+	3.85 × 10^−3^
10621	+	5.56 × 10^−2^	−		+	5.92 × 10^−5^
10644	+	2.40 × 10^−2^	−		+/−	
10645	+	2.03 × 10^−1^	−		+	2.39 × 10^−5^
10647	+	1.46 × 10^−4^	−		+	1.43 × 10^−2^
10649	+	7.44 × 10^−6^	+	2.57 × 10^−7^	+	1.54 × 10^−4^
10678	+/−		−		−	
10682	+/−		−		−	
10693	+	1.46 × 10^−4^	+	2.28 × 10^−6^	+	5.17 × 10^−5^
10754	+	6.37 × 10^−6^	+	1.22 × 10^−6^	+	1.66 × 10^−5^
10811	+	8.54 × 10^−3^	+/−		+	6.58 × 10^−7^
10856	+	4.88 × 10^−5^	+/−		+	7.89 × 10^−4^
10934	+	1.68 × 10^−3^	+/−			
10939	+	9.76 × 10^−5^	+/−		+/−	
10940	+	3.41 × 10^−4^	−		−	
12101	+	2.80 × 10^−3^	+/−		−	
12104	+	1.00 × 10^−2^	+	5.95 × 10^−6^	+/−	
12105	+	1.06 × 10^−2^	+	1.19 × 10^−4^	+	2.39 × 10^−3^
12108	+/−		−		+	7.50 × 10^−6^
12109	+	1.30 × 10^−2^	+	1.34 × 10^−5^	+	9.54 × 10^−5^
12110	+	4.32 × 10^−4^	+	3.73 × 10^−5^	+	3.29 × 10^−6^
12111	+/−		+/−		−	
12112	+/−		+/−		−	
12113	+	1.93 × 10^−2^	+	3.27 × 10^−5^	+	6.91 × 10^−7^
12114	+	9.76 × 10^−4^	+	8.65 × 10^−7^	−	
12115	+	6.34 × 10^−4^	+	2.49 × 10^−6^	+	5.92 × 10^−6^
12116	+	3.27 × 10^−2^	+/−		+	
12117	+	1.52 × 10^−2^	+	1.25 × 10^−7^	+	1.02 × 10^−5^
12118	+	2.63 × 10^−2^	+	7.49 × 10^−6^	+	1.91 × 10^−5^
12119	+	8.88 × 10^−5^	+	2.11 × 10^−7^	+	1.61 × 10^−6^
12120	+/−		+/−		−	
12121	+/−		+/−		+/−	
12122	+	2.03 × 10^−1^	+	9.01 × 10^−5^	+	3.81 × 10^−3^
12123	+	8.56 × 10^−3^	+	6.33 × 10^−6^	+	4.27 × 10^−4^
12124	+/−		+/−		−	
12125	+	2.46 × 10^−3^	+	6.54 × 10^−3^	+	4.64 × 10^−2^
12126	+	6.39 × 10^−3^	+	1.58 × 10^−6^	+	3.03 × 10^−6^
12128	+/−		+/−		−	
12134	+	2.78 × 10^−3^	−		−	

^a^ Spot test results: (+) sensitive for phage; (−) not sensitive for phage; (+/−) unclear result. ^b^ EOP: relative “efficiency of plating”; relative to the highest phage concentration (pfu/mL) obtained for the respective propagation strain (^c^ ST29: 4.1 × 10^9^ pfu/mL, ^d^: ST11: 4.7 × 10^10^ pfu/mL and ^e^ ST27: 1.5 × 10^10^ pfu/mL). Calculated by dividing the phage concentration measured on the test strain by the phage concentration on the respective propagation strain.

**Table 2:** Bacteriophages and propagation strains.

Phage	Phage Origin	Details	Propagation Strain (*S. aureus*)	Strain Origin
STA1.ST29	sewage water	Myovirus; related to phage K	ST29	human isolate
STA1.ST107	sewage water	Myovirus; related to phage K	ST107	pig farm
EB1.ST11	pig manure	Podovirus related to phage PSa3	ST11	mastitis milk sample
EB1.ST27	pig manure	Podovirus related to phage PSa3	ST27	mastitis milk sample

All phages were isolated and sequenced by PTC GmbH, Bönen, Germany.
